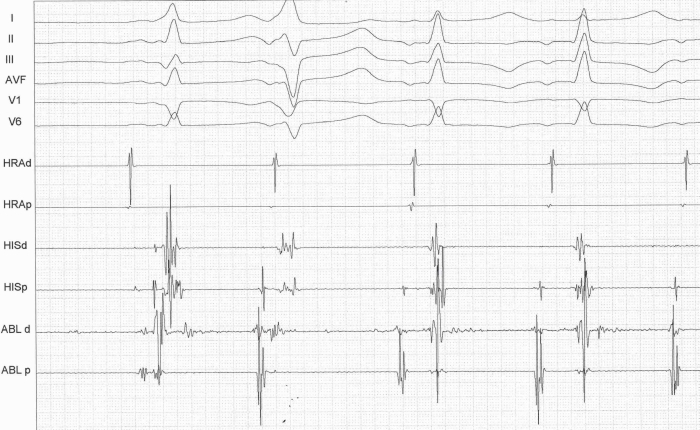# Development of Malignant Ventricular Arrhythmias in a Young Male with WPW Pattern

**Published:** 2010-04-01

**Authors:** Alim Erdem, Nihat Madak, Ahmet Yilmaz, Osman Can Yontar, Hasan Yucel, Ibrahim Gul, Izzet Tandogan

**Affiliations:** 1 Cardiology Department, Sivas Public Hospital, Kars, Turkey; 2 Cardiology Department, Malatya Public Hospital, Malatya, Turkey; 3 Cardiology Department, School of Medicine, Cumhuriyet University, Sivas, Turkey

**Keywords:** WPW syndrome, life threatening arrhythmias

## Abstract

In Wolff-Parkinson-White Syndrome (WPW), presence of accessory pathways causes various tachyarrhythmias that lead to different symptoms and clinical conditions in patients. Atrial fibrillation is observed in about 20-30% of this group of patients. Life threatening malignant ventricular arrhythmias and sudden cardiac deaths are observed in patients having rapid conduction in accessory pathways and short antegrade refractory periods (<250 msn). We present a WPW syndrome case that presented to the emergency service with narrow QRS tachycardia and later developed malignant ventricular arrhythmia.

## Case Report

**Figure 1:**
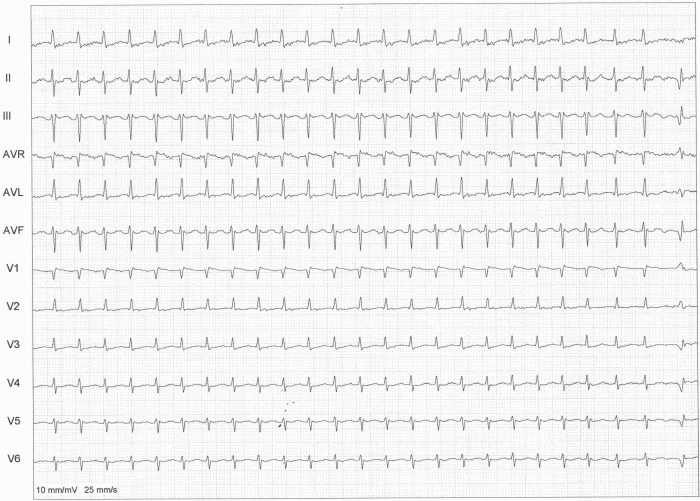
Narrow QRS complex tachycardia at presentation

**Figure 2:**
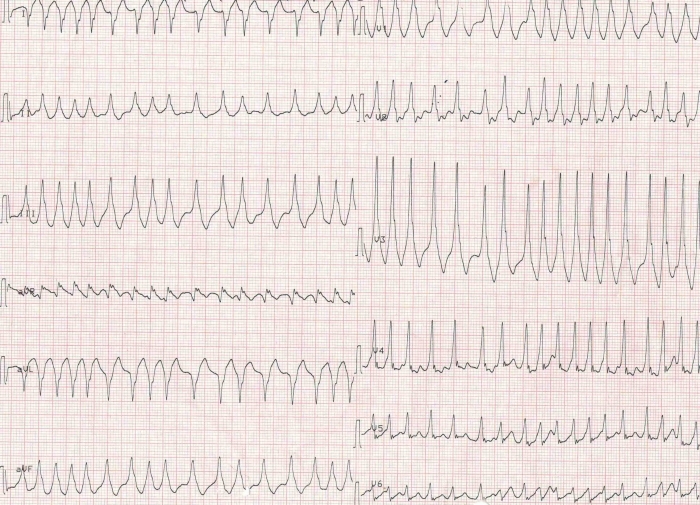
Atrial fibrillation with wide QRS and high ventricular response

**Figure 3:**
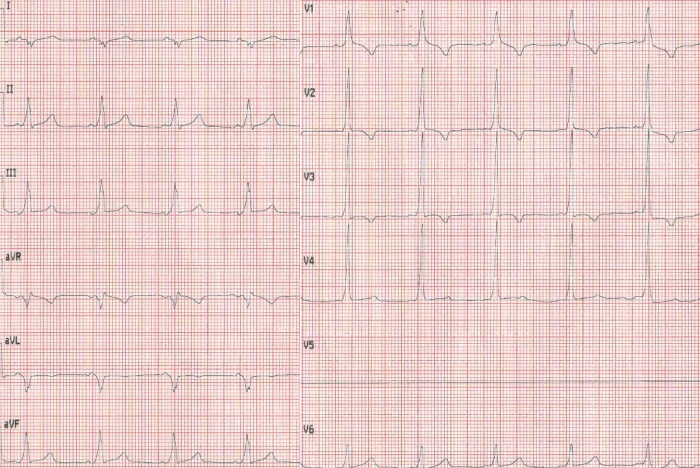
12 lead ECG showing characterstic delta waves and short PR interval of WPW syndrome

**Figure 4:**
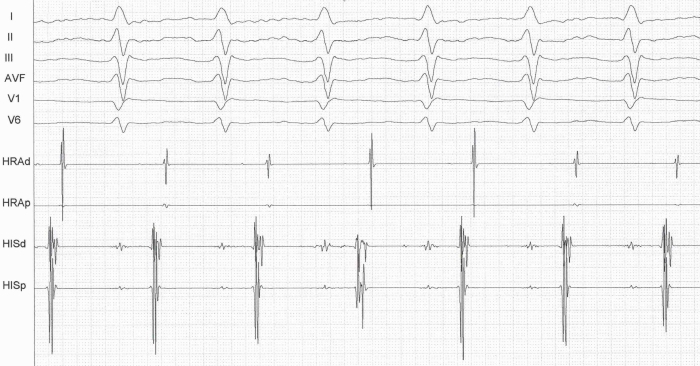
Orthodromic tachycardia induced during EPS

A 32 years old male with no previous cardiac evaluation and clinical complaints presented to the emergency service with palpitation. Due to narrow QRS complex tachycardia, emergency service physicians (Figure 1) administered intravenous verapamil for rate control. Meanwhile, the patient developed atrial fibrillation with wide QRS and high ventricular response (Figure 2) and then ventricular fibrillation. Normal sinus rhythm was achieved after multiple defibrillations (220 joule, Biphasic). Hemodynamic parameters and respiration were stabilized. 12 lead ECG showed characterstic delta waves and short PR interval. (Figure 3). Given these findings, the case was diagnosed as Wolff-Parkinson-White (WPW) syndrome. His physical examination, biochemical parameters and echocardiography were normal. As the case was a WPW syndrome case and as malignant ventricular arrhythmia and cardiopulmonary arrest developed due to atrial fibrillation, he was assigned to electrophysiological (EPS) study. In our case, whose orthodromic tachycardia was induced again during EPS (Figure 4). An accessory pathway was found at left anterior localization based on intracardiac findings, and successful ablation was achieved in the same session (Figure 5). On the surface ECG, it was observed that PR interval was back to normal and delta wave was no more present. The case was asymptomatic and his ECG was normal during his controls at 1st and 6th months.

## Discussion

WPW syndrome is a clinical entity characterized by presence of an electrical signaling accessory pathway between atrium and ventricular that may cause tachyarrhythmia's sometimes and sudden cardiac death. Studies have shown that SCD occurs at the rate of 0.15% per year in patients with WPW. These deaths are caused by atrial fibrillation with rapid ventricular response that leads to ventricular fibrillation (VF) [1,2].

The most frequently encountered tachycardia in WPW syndrome is the reentrant tachycardia. The degeneration of reciprocating tachycardia to atrial fibrillation is not uncommon [3]. It has been estimated that atrial fibrillation (AF) is observed in approximately one third of WPW patients. AF may cause fatal arrhythmias in WPW syndrome [4]. Sudden death occurs due to transmission of AF into VF with a rapid ventricular response over one or more accessory pathways with a short antegrade refractory period [5]. The first presentation of some WPW patients with asymptomatic clinical course could be due to ventricular fibrillation [6].

Risks factors for sudden death in WPW are presence of more than one AP, development of AVRT along with AF and the shortest preexcitation RR interval less than 260 msn [1,2,7]. Development of atrial fibrillation as a result of  IV verapamil administration for rate control in reciprocal tachycardia is not rare [8]. Straberg et al reported 3 cases presented with  atrial fibrillation and rapid ventricular response with wide preexcitation QRS, all of whom received intravenous verapamil (5-10 mg); and they stated that one of the patients developed ventricular fibrillation requiring several defibrillations, the other had hemodynamic deterioration and the last one had a  marked increment in the ventricular response. When verapamil is used to treat reentrant supraventricular tachycardia complicating the WPW syndrome, the electrophysiologic effects of verapamil on accessory pathway conduction during atrial fibrillation become critical. Patients with WPW syndrome have a higher incidence of atrial fibrillation than the general population [10,11]. We think that verapamil administered for rhythm and rate control at emergency service induced AF with wide QRS and consequent VF development in our case too. Antz et al. stated that patients with WPW syndrome who had undergone CPR for malignant ventricular function had normal left ventricular function at echocardiography and no ECG abnormalities and ablation of their accessory pathways prevented cardiac arrest recurrences [12]. Catheter ablation is suggested for patients resuscitated from VF or for patients at high risk for clinical atrial fibrillation with a rapid ventricular response [13].

As a result, Using AV node suppressive medicines in patients with WPW syndrome may cause atrial fibrillation followed by development of fatal cardiac arrhythmias. We believe that it is important to acquaint all the clinicians, especially the emergency service and ambulance physicians who are the first to face SVT patients in daily practice, with this issue.

## Figures and Tables

**Figure 5:**